# Predicting the Phenotypic Values of Physiological Traits Using SNP Genotype and Gene Expression Data in Mice

**DOI:** 10.1371/journal.pone.0115532

**Published:** 2014-12-26

**Authors:** Yu Takagi, Hirokazu Matsuda, Yukio Taniguchi, Hiroaki Iwaisaki

**Affiliations:** Graduate School of Agriculture, Kyoto University, Kyoto 606-8502, Japan; University of Illinois at Chicago, United States of America

## Abstract

Predicting phenotypes using genome-wide genetic variation and gene expression data is useful in several fields, such as human biology and medicine, as well as in crop and livestock breeding. However, for phenotype prediction using gene expression data for mammals, studies remain scarce, as the available data on gene expression profiling are currently limited. By integrating a few sources of relevant data that are available in mice, this study investigated the accuracy of phenotype prediction for several physiological traits. Gene expression data from two tissues as well as single nucleotide polymorphisms (SNPs) were used. For the studied traits, the variance of the effects of the expression levels was more likely to differ among the genes than were the effects of SNPs. For the glucose concentration, the total cholesterol amount, and the total tidal volume, the accuracy by cross validation tended to be higher when the gene expression data rather than the SNP genotype data were used, and a statistically significant increase in the accuracy was obtained when the gene expression data from the liver were used alone or jointly with the SNP genotype data. For these traits, there were no additional gains in accuracy from using the gene expression data of both the liver and lung compared to that of individual use. The accuracy of prediction using genes that were selected differently was examined; the use of genes with a higher tissue specificity tended to result in an accuracy that was similar to or greater than that associated with the use of all of the available genes for traits such as the glucose concentration and total cholesterol amount. Although relatively few animals were evaluated, the current results suggest that gene expression levels could be used as explanatory variables. However, further studies are essential to confirm our findings using additional animal samples.

## Introduction

Recent advances in high-throughput technologies have generated large amounts of single nucleotide polymorphism (SNP) data in many species. In addition, gene expression data are gradually becoming available. In humans, gene expression data have been used in clinical diagnosis and prognosis prediction [1]–[4]. Additionally, the use of gene expression data has increased the accuracy of predicting the phenotypic values of complex traits in some species [5]–[8]. As previously emphasized [9], [10], the prediction of phenotypes is useful in a wide range of fields, such as evolution, medicine, human biology, and crop and livestock breeding.

SNP and gene expression data may be used for genome-wide association studies (GWAS). In GWAS, it is usually expected that trait-associated SNPs coincide with the causal variants. However, linkage disequilibrium in the region of interest often makes it difficult to simply identify the causal variants that affect a trait of concern. Although the detection of genes with trait-correlated expression levels is relatively easy, there is no guarantee that the association causes the phenotype. Therefore, the joint use of both SNP and gene expression data is expected to improve the accuracy and robustness of the prediction of phenotypic values for complex traits because the advantages of SNP data include compensating for the shortcomings of gene expression data and vice versa.

The pioneering work for the prediction of phenotypes using both SNP genotype and gene expression data involved the drug response in yeast (*Saccharomyces cerevisiae*) and was performed by Chen et al. [5], who showed a greater accuracy of the prediction using both types of data. Chen et al. thus developed a causal model with expression linkage for complex traits (Camelot) that considered the causal relationship between the variables. Recently, for the pathogen resistance of soybeans, Bhattacharjee and Sillanpaa (2011) [7] showed that the prediction accuracy improved via the selected use of explanatory variables that were composed of SNP genotypes and gene expression levels; these authors implemented a Bayesian estimation [11] and supervised a principal component analysis [12]. It is important to note that neither of these studies used gene expression information that had been acquired in the physiological state that was directly related to the trait of concern. That is, Chen et al. [5] used information on gene expression for yeast under drug-free conditions, and Bhattacharjee and Sillanpaa (2011) [7] used expression information from soybeans that were not infected with a pathogen. Although these aspects suggest the valid use of gene expression data in predicting complex traits, it is necessary to further examine whether a positive result obtained in these studies could be applicable to other species, such as mammals [13].

In a multicellular organism, gene expression profiles differ in each tissue or cell; therefore, the profiles that are most relevant to the trait of concern are often unavailable [14]–[17]. When blood samples or cell lines are used to measure gene expression levels, the applicability of the obtained data to other tissues should be investigated. To date, only two studies using both transcript and SNP genotype data in mice have been conducted [8], [18], and the gene expression data were only obtained from liver samples. Even in mice, the data sources that are available for the prediction of phenotypes using gene expression information are still very limited, and their sample sizes are small. It is therefore necessary to collate the obtained results using available sources of gene expression data.

In this report, by targeting certain physiological traits in mice and employing different statistical methods for prediction, we assess the profiles of prediction accuracy for phenotypes using gene expression data in two tissues as well as SNP genotype data.

## Materials and Methods

### Sample data and studied traits

The data that were used were phenotypic values of physiological quantitative traits, genome-wide SNP genotypes, gene expression levels in the liver and lung in heterogeneous stock (HS) mice, and gene expression levels in several organs of C57BL6 mice; the former two types of data were collected from the database of the Wellcome Trust Centre for Human Genetics (http://gscan.well.ox.ac.uk/). The HS mice originated from a crossing of eight inbred strains (A/J, AKR/J, BALBc/J, CBA/J, C3H/HeJ, C57BL/6J, DBA/2J, and LP/J) followed by 50 generations of pseudorandom mating, and the dataset of phenotypic records was represented by full-sib families. Among the 12,545 SNPs that were polymorphic in at least some of the eight inbred strains and that were genotyped by the Illumina custom BeadArray platform [19], a total of 11,037 SNPs were used for each mouse, excluding those with a minor allele frequency of <0.05 and a call rate of <0.90.

We attempted to use the gene expression data from mice that provided data from the liver and lung tissues normalized by the variance stabilization method of Huber et al. [20], specifically focusing on experiment ID E-MTAB-88 [21] in the ArrayExpress database (http://www.ebi.ac.uk/arrayexpress/). The expression data were obtained using the Illumina Mouse-6 v1 Expression BeadArray (Illumina, Inc., San Diego, CA, USA) containing 47,429 unique probe sequences. The expression levels of each gene were standardized to have mean 0 and variance 1, as described by Chen et al. [5]. Because the mouse chips that were used in the array experiments often contained two or more probes corresponding to the same gene, the redundant probes were removed from the dataset using the highest minimal method [4]. Consequently, a total of 33,310 annotated probes, 23,856 of which code for a protein or have a known mRNA reference sequence, were selected for use. Although alternative splicing events were not considered in this study, a more-precise analysis including alternative mRNA splicing could be implemented by using a re-annotation of the Illumina probe sequences [22]. The microarray platforms that contain multiple probes per exon, such as Affymetrix exon arrays, are substantially more useful in distinguishing between different isoforms of a gene.

The traits that were studied included 5 physiological traits: blood glucose, insulin, and total hemoglobin concentrations; total cholesterol content; and tidal minute volume. Because we could use gene expression data only from the liver and lung, among the traits that we could study, the blood glucose and insulin concentrations and total cholesterol content were assumed to be related to the liver, while the total hemoglobin concentration and tidal minute volume were assumed to be related to the lung. The details for measuring these traits are given in Solberg et al. [23]. The numbers of mice that were used in the analyses of the glucose, insulin, and hemoglobin concentrations; total cholesterol amount; and tidal minute volume were 242, 241, 209, 232 and 251, respectively, for which the gene expression levels in the liver were available, and 251, 249, 213, 241, and 260, respectively, for which the gene expression levels in the lung were available.

### Gene selection

In this study, the selective use of genes and the use of all of the genes in the prediction of phenotypic values were conducted in three ways.

One criterion for the selection was the significance level of the effect of gene expression on the full-sib family variation. A one-way analysis of variance for the gene expression levels after the standardization of the genes was conducted, in which the two factors were full-sib family group and residual. The genes that were selected for use consisted of the top genes with relatively high and low significance levels (3,000 each; denoted as SL higher and SL lower, respectively). We expected that the selected genes would reflect a hereditary effect on the gene expression levels. Another criterion was whether the genes were significantly expressed in a specific tissue (liver or lung). To determine the genes for selection in this manner, the information of the gene expression levels that were reported in several organs of C57BL6 mice [24] was used. The median gene expression levels for gene j across all of the tissues (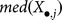) were calculated, and the tissue-specific value was defined as
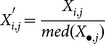



where 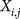 is the expression value of gene j at tissue i, and 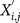 is the tissue-specific value. Then, the top 3,000 genes with high values (TS higher) were selected. The third criterion was the magnitude of the standard deviation in the expression levels. Using the gene expression levels before standardization, the standard deviation for every gene was assessed, and the top 3,000 genes with higher standard deviations (SD higher) were selected. The genes that were used to predict the phenotypic values are summarized in Table 1.

**Table 1:** Criteria and rationales for gene selection.

Genes for use	Criterion	Rationale
SL higher (lower) genes	Significance level of the among-family variance by one-way ANOVA	The hereditary effect on the gene expression levels might affect prediction accuracy when both the SNP genotypes and gene expression levels are used.
TS higher genes	Tissue-specific value	Highly expressed genes in the target tissue are likely to be associated with the phenotypic value.
SD higher genes	Standard deviation on the original scale	Equally expressed genes should not be strongly associated with the phenotypic value.

### Statistical methods for prediction

Bayesian regression methods have been applied to large-p-small-n data (e.g., [7]; [25]). Bayesian ridge regression assumes that effect of each marker follows a normal distribution with equal variance across all of the markers. In addition, Bayesian lasso regression [26], which is a Bayesian counterpart of the Least Absolute Shrinkage and Selection Operator (LASSO; [27]), may be a better choice than the Bayesian ridge regression if most markers have a relatively small effect and only a few markers have a sizable effect because it produces a marker-specific shrinkage of effects. In this study, the Bayesian ridge and lasso regressions were used to estimate the effects of the SNP genotype and gene expression level on the phenotype. The linear model that was used to describe the phenotypes was 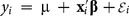, where 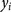 is the phenotypic value of the *i*th individual, 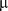 is the overall mean, 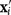 is the vector of the explanatory variables; for SNPs, the genotype codes based on the number of the minor allele for each SNP were used, 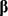 is the vector of the regression coefficients, and 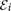 is the residual with variance 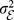.

The regression coefficients 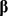 were assigned identical-independent Gaussian priors with the variance 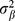 that is common to all of the effects for the Bayesian ridge regression and identical-independent double-exponential (i.e., Laplace) priors for the lasso regression, in which a double-exponential distribution can be written as a scaled mixture of normal densities with a different variance in every effect [26]. The prior variance of the lasso induces the marker-specific shrinkage of the estimates, and the extent of the shrinkage depends on the scale parameter that was assigned, the exponential prior and the regularization parameter that was assigned a gamma prior with rate and shape parameters equal to 0.001. The variance parameters of 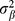 and 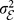 were assigned independent scaled inverted chi-square distributions with the degree of belief and scale parameters of -2 and 0, respectively.

### Cross validation

In the preliminary analysis, we compared 5-fold and 10-fold cross-validations, and the results were matched (data not shown). Therefore, a 5-fold cross validation was conducted to save computing time to evaluate the prediction accuracy. Because the phenotypic records that were used in this study were represented by full-sib families, five subsets of the data were created by dividing all of the families into five groups. The regression analyses for the prediction of phenotypic values were conducted using four of the five subsets as training data. Then, for the obtained prediction equation, the accuracy of prediction was computed using the remaining subset as the test data. The accuracy was evaluated based on the average of five values for the correlation between the predicted and actual phenotypic values.

There were dependencies between the correlations because of common individuals in the training data. For this type of setting, a statistical test was not performed in most of the previous reports that investigated differences in the accuracy of genomic evaluation by cross-validation, for instance, with different prediction methods. The likely reason for this lack of statistical testing is that the confidence intervals for the accuracies often highly overlap, which gives the false impression of statistical non-significance. The Hotelling-Williams t-test [28] considers a strong correlation between the predictions from different methods or approaches and, therefore, may be a relatively powerful test [29]. However, this test requires the same animals for cross validation. In this study, this requirement was not always satisfied so that the maximum number of animals could be used, as the total number of animals available was limited. Thus, according to previous studies, the differences in the means of the correlations in this study were tested for statistical significance by the paired t-test.

For the gene selections based on the SL higher, SL lower, and SD higher criteria, we used only the training data so that the information in the test data did not affect the gene selections. As stated above, the gene expression data [24] that were used to select the TS higher genes were independent of the HS mice data [21].

### Computation

All of the analyses with either the ridge or lasso regression methods were implemented via Gibbs sampling using the R package BLR [30]. In total, 25,000 cycles of Markov chain Monte Carlo (MCMC) simulations were implemented, and the values of the unknown parameters were sampled every 10 cycles during the last 20,000 cycles after the first 5,000 cycles were discarded as burn-in. The convergence was checked via the graphical evaluation of the trace plots. The posterior samples for the regression coefficients from the MCMC cycles were used to estimate the variances that were explained by the SNPs and gene expression levels as described by Ehsani et al. [8]. While all of the markers have a common variance in the Bayesian ridge regression, in the lasso regression, the variables are weighted by the scale parameter, and all of the markers have an effect, but the variances of the effects are still not the same.

## Results and Discussion

### Goodness-of-fit for statistical methods

**Figure 1:**
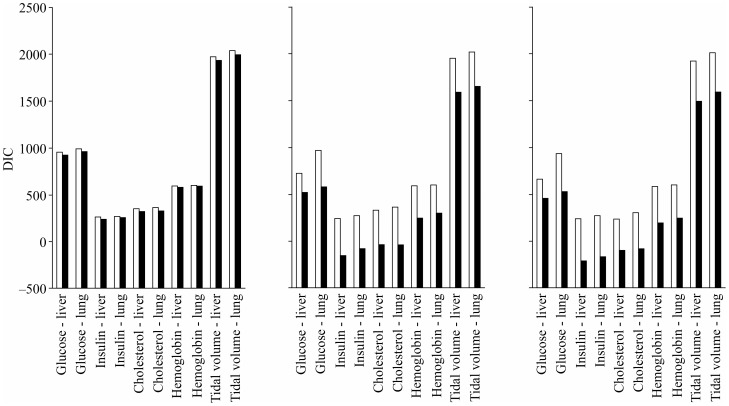
Comparison of the DICs between the Bayesian ridge and lasso regression methods. The DIC values were obtained using SNP genotypes (left), gene expression levels (middle), and both SNP genotypes and gene expression levels (right) as explanatory variables. The white and black bars correspond to the Bayesian ridge and lasso regression methods, respectively.

The deviance information criteria (DIC) [31] that were obtained with the ridge and lasso regressions are shown in Fig. 1 for the cases where the SNP genotype and gene expression level were used as explanatory variables. When the SNP genotype was used as an explanatory variable, there were no substantial differences in the DIC values between the two methods for every case or in the combinations of traits and tissues in which the expression levels of genes were used. In contrast, when the gene expression levels were used, the value of the DIC with the lasso was obviously lower than that with the ridge regression for every case, demonstrating that the use of the former method provided better fits for all of the traits. Lower DIC values with the lasso regression were also observed in the cases where both the SNP genotype and gene expression level were used together. The lasso method may have provided a better fit partly because this method may consider heterogeneous effects on gene expression, as previously suggested [32]. Moreover, for the traits that were studied here, it is likely that the effects of the expression levels differ among the genes even though the variances of the SNP effects are relatively homogeneous.

### Variances explained

The estimates of variances due to the SNP genotype and gene expression level with the lasso method are shown in Table 2 for the three selected traits. Approximately 40% to 50% of the phenotypic variance was explained by the SNP genotype for the traits. Using approximately 2,000 records and more than 10,000 SNP genotype data on the same HS mouse population as in this study, Valdar et al. [33] obtained heritability estimates of 0.55, 0.38, and 0.17 for the glucose concentration, total cholesterol amount, and tidal minute volume, respectively. The estimated proportions of the variance due to the SNPs to the phenotypic variance in this study were similar to the heritability estimates for the glucose and total cholesterol contents, although the current estimate for the tidal minute volume was higher than these estimates. In contrast, the gene expression levels explained approximately 65% to 70% of the phenotypic variance for the three traits irrespective of the tissue. Only Ehsani et al. [8] previously estimated the variances due to the SNP genotype and gene expression using 440 individuals in a different mouse population, although the studied traits were the body weight, feed intake, and feeding efficiency. The current attribution of a higher proportion of the estimated variance to the gene expression level than to the SNP genotypes agrees with the findings of Ehsani et al. [8].

**Table 2:** Variance in three traits as explained by the SNP genotype and gene expression level using the Bayesian lasso regression.

			Explanatory variable					
Trait	Tissue^a^	Variance^b^	SNP genotype (S)		Gene expression level (G)		S + G^d^	
			Estimate^c^	Percentage	Estimate	Percentage	Estimate	Percentage
Glucose (mg/dl)	Liver	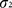	4.86 (0.53)	54	2.72 (2.19)	31	0.24 (0.08)	3
		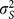	4.08 (0.61)	46	-		1.38 (0.20)	16
		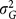	-		6.00 (1.99)	69	6.87 (0.40)	81
		Total	8.94		8.72		8.49	
	Lung	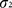	4.79 (0.53)	53	2.85 (2.00)	32	0.39 (0.08)	5
		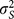	4.21 (0.61)	47	-		1.56 (0.22)	18
		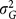	-		5.96 (1.83)	68	6.69 (0.39)	77
		Total	9.00		8.81		8.65	
Total cholesterol (mmol)	Liver	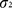	0.23 (0.03)	54	0.13 (0.10)	31	0.02 (0.003)	4
		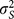	0.20 (0.03)	46	-		0.08 (0.01)	19
		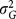	-		0.29 (0.09)	69	0.32 (0.02)	77
		Total	0.44		0.43		0.39	
	Lung	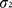	0.23 (0.03)	53	0.13 (0.10)	32	0.02 (0.004)	5
		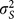	0.20 (0.03)	47	-		0.08 (0.01)	19
		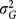	-		0.29 (0.09)	68	0.31 (0.02)	76
		Total	0.43		0.42		0.41	
Tidal minute volume	Liver	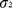	691.4 (68.7)	60	392.3 (305.3)	35	45.0 (10.1)	4
(baseline)		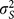	469.5 (70.6)	40	-		194.3 (28.6)	17
		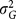	-		735.2 (273.4)	65	878.4 (47.3)	79
		Total	1160.9		1127.5		1117.7	
	Lung	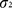	675.5 (68.6)	59	389.1 (292.6)	35	44.3 (7.55)	4
		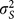	472.9 (72.3)	41	-		193.5 (27.3)	18
		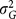	-		729.2 (264.4)	65	861.3 (47.2)	78
		Total	1148.4		1118.3		1099.1	

a Tissue in which gene expression levels were measured.

b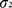, 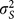, and 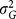 denote the residual variance and the variance due to the SNP genotype and gene expression levels, respectively.

c Posterior standard deviation in parentheses.

d Both the SNP genotype and gene expression level as explanatory variables.

When the gene expression level and SNP genotype were used jointly as explanatory variables, more of the total variance (>90%) was explained, with relatively high variance attributed to the gene expression level. Although we confirmed the convergence in the estimation, an overestimation of the variance may have occurred, particularly in the joint use of the SNP genotype and gene expression level because the number of animals providing gene expression data in this study was limited and was well exceeded by the number of SNPs and gene expression levels.

### Accuracy of the predicted phenotype

**Figure 2:**
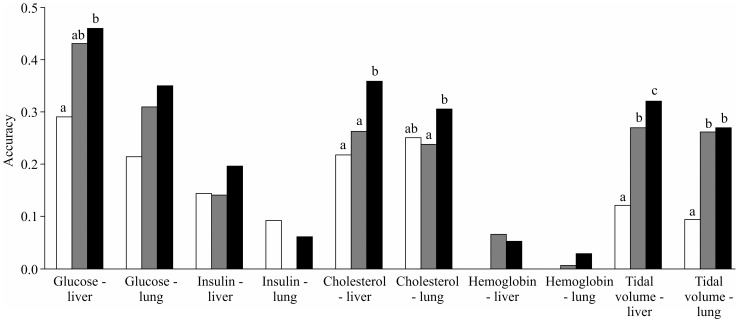
Accuracy of prediction with the Bayesian lasso regression. The bars sharing the same letter are not statistically significant (P ≥ 0.05) according to a paired t-test. The term ‘glucose - liver’ on the x-axis represents the prediction of the glucose concentration using the gene expression levels in the liver. The white, gray, and black bars correspond to the accuracy of prediction using the SNP genotype, gene expression level, and both SNP genotype and gene expression level, respectively.

The accuracy of the predicted phenotypic values using the SNP genotype and gene expression level as explanatory variables is shown in Fig. 2 for the lasso regression. Although the proportion of the variance that was explained by the SNP genotype and gene expression level to the total variance was approximately >50%, the accuracy of prediction in the cross-validation study was considerably low, which is a typical observation in genomic predictions with ‘the p> n problem’, where the number of explanatory variables (p) exceeds the number of independent variables (n). The accuracy with the ridge was similar to that with the lasso when the SNP genotypes were used, but the ridge accuracy was lower than the lasso accuracy when the gene expression levels were used (data not shown).

When the SNP genotypes were used in the lasso regression, the accuracy was in the range of approximately 0.3 to 0.2 for the glucose concentration (mg/dl) and total cholesterol content (mmol), followed by values of approximately 0.15 to 0.1 for the insulin concentration (ng/mL) and tidal minute volume (baseline). The accuracy was essentially zero for the hemoglobin concentration (g/dL). Compared to these accuracy values, when the gene expression levels were used in place of the SNP genotypes, the accuracy tended to increase by approximately 0.1 for the glucose concentration and by approximately 0.1 to 0.25 for the tidal minute volume, although the differences did not reach significance, possibly because of the number of records that were available in this study. For the use of both the SNP and gene expression data, the accuracy tended to further increase, although there were a few exceptions in the cases of the traits that showed a low accuracy below 0.1. For the glucose concentration and total cholesterol content, even though relatively few animals were used in this study, a significant difference in the accuracy was observed between the use of the SNP genotypes and that of the gene expression levels in the liver in addition to the SNP genotypes. These results suggest that the gene expression levels in the tissue related more strongly to the target trait, improving the accuracy of the prediction.

For the tidal minute volume, which we may simply believe would have a relatively strong and weak relationship with the lung and liver, respectively, the accuracy of prediction was relatively low when using the SNP genotypes but increased significantly (P <0.05) when the gene expression levels in the lung were used. This observation may indicate that the gene expression data reflect effects that are not explained by the SNP data. Furthermore, for this trait, the accuracy of prediction using gene expression levels in the liver also increased significantly (P <0.05) when compared with the use of the SNP genotypes. We calculated the phenotypic correlations in 183 animals that had phenotypic values for all of the traits. While most of the estimates were very low to low, the estimated correlation between the tidal minute volume and the glucose concentration was sizable and relatively high, with a value of 0.23. This observation may suggest that although the magnitude is low, the liver, which is involved in glucose metabolism, might be indirectly related to the gene expression and tidal minute volume through blood flow, given the widespread influence of liver function. The approach of Hu et al. [4] may be used to further investigate the possibility of such an indirect relationship.

For the hemoglobin concentration, all of the predictions using either the SNP genotypes or gene expression levels produced very low values of accuracy. We expected that the gene expression levels in the lung would substantially contribute to the variation in the hemoglobin concentration. However, the accuracy of prediction using the expression levels in the lung was very low for this trait, suggesting that, in fact, the relationship between the gene expression in the lung and the hemoglobin concentration is weak.

For the insulin concentration, the accuracy of prediction using the gene expression levels in the lung was also very low. Although there are various regulatory pathways in which the lung influences the level of blood insulin in the endocrine network (EndoNet database; http://endonet.bioinf.med.uni-goettingen.de/; [34]), these pathways may be less likely to have large effects on the phenotypic variation. Additionally, because these pathways are intermediated by multiple tissues and organs, including adipose tissue and the pancreas, it is also possible that the lung expression data do not sufficiently reflect the influences of these pathways. For the glucose concentration and total cholesterol content, however, the observed accuracy was relatively high when the gene expression levels in the lung were used. Path analysis by EndoNet also shows that glucose metabolic process and homeostasis are affected by the lung. However, the path from the lung to cholesterol does not exist in the database; therefore, the results for the total cholesterol content cannot simply be explained by known intercellular regulatory relationships. Although an analysis of the ontogenetic relationships between mammalian tissues revealed that the liver and lung are developmentally close [35], Yanai et al. [36] suggested that even when development or functions are similar between tissues, there is no evidence that the gene expression levels are similar. In fact, the gene expression levels in the liver and lung that we used in this study had essentially zero correlation. The accuracy of the prediction of the glucose concentration and total cholesterol content using the SNP genotype data was relatively high in this study, and the heritability estimates were approximately 0.5 in the study of Valdar et al. [33] and in the current study. These findings suggest that the hereditary effects of the gene expression levels improved the accuracy of prediction. The expression quantitative trait locus (eQTL) analyses revealed a tendency for eQTLs to be common among different tissues in an organism, and as many as 70% of the cis-eQTLs have been reported to be common among tissues [37]. Therefore, the gene expression information from tissues or cells that are not related directly to the trait of concern may still be useful as markers for the trait.

### Integrated use of the gene expression data

**Figure 3:**
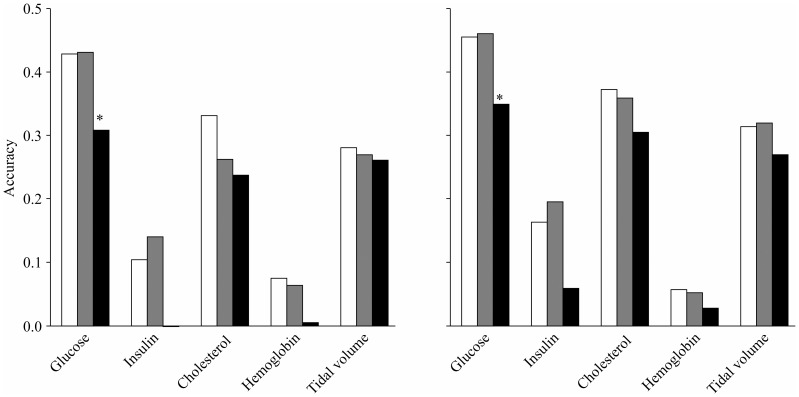
Accuracy of prediction jointly using the gene expression levels in the liver and lung (white) compared to the accuracy using the gene expression levels in the liver (gray) or lung (black). The left and right panels show the accuracy using the gene expression level alone and both the SNP genotype and gene expression level together, respectively. The bar that is labeled with an asterisk is significantly different (P <0.05) from the white bar according to a paired t-test.

Fig. 3 presents the accuracy of prediction with the lasso method jointly using the gene expression levels in the liver and lung compared to using either type of expression data. Overall, the accuracy using the gene expression levels in the lung was relatively low. In almost all of the cases, the accuracy using the gene expression levels in both tissue types was similar to the corresponding accuracy in the liver, even when a model that did not include the SNP effects as explanatory variables was fit. These results suggest that for the traits that were investigated in this study, there may be no additional benefits of the integrated use of gene expression information in the liver and lung as explanatory variables, although it is unknown whether this result can be generalized to other tissues.

### Use of selected genes

**Figure 4:**
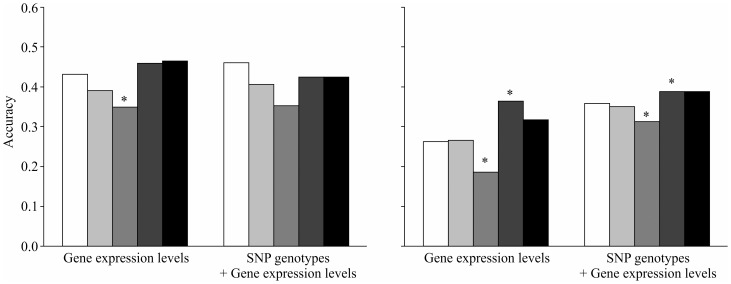
Influence of gene selections on the accuracy of prediction. The accuracy of prediction for the glucose concentration (left) and total cholesterol amount (right) with the lasso method using the expression levels of all of the genes and selected genes in liver. The ‘gene expression levels’ and ‘SNP genotypes + gene expression levels’ on the x-axis represent the accuracy using the gene expression levels alone and using both the SNP genotypes and gene expression levels together, respectively. The white, light gray, gray, dark gray, and black bars correspond to the accuracy using all of genes, the SL higher genes, the SL lower genes, the TS higher genes, and the SD higher genes, respectively. The bar that is labeled with an asterisk is significantly different (P <0.05) from the white bar according to a paired t-test.

Fig. 4 shows the accuracy of the prediction of the glucose concentration and total cholesterol content with the lasso method using the expression levels of all of the genes and selected genes in the liver. When only using the gene expression levels, the accuracy tended to be lower than or similar to that using all of the genes when the SL higher genes were used, whereas the accuracy was significantly decreased for both of the traits when the SL lower genes were used.

The use of the TS higher genes to predict the glucose concentration and total cholesterol content resulted in accuracies that were similar to and greater than that obtained using all of the genes, respectively, where the increase in the total cholesterol content was statistically significant. There was a similar tendency for an approximately equal or greater accuracy when using the TS higher genes for traits other than the glucose concentration and total cholesterol, although the differences were not statistically significant (data not shown). Additionally, the prediction using the SD higher genes tended to produce at least a similar level of accuracy as that obtained when using of all of the genes. This finding is important because the number of explanatory variables that were used was 3,000, which was much lower than the total number of available gene expression levels (24,000) in this study, although the number of explanatory variables still exceeded the number of animals that were used.

In the case of the TS higher genes, we performed a Gene Ontology (GO) enrichment analysis to compare the 3,000 selected genes with all of the genes in the liver by Fisher's exact test using the Database for Annotation Visualization and Integrated Discovery (DAVID) tool [21]. The significant GO terms (Bonferroni <0.05) are listed in Table 3. The TS higher genes in the liver were enriched in GO terms, including the cholesterol metabolic process and glucose metabolic process, supporting the current prediction results.

**Table 3:** Representative GO terms (biological process) for the comparison between the TS higher genes and all of the genes in the liver.

GO ID	GO biological process	Count of genes	P-value	Bonferroni^a^
GO:0051186	cofactor metabolic process	90	4.5E-27	1.8E-23
GO:0008202	steroid metabolic process	85	1.4E-26	5.6E-23
GO:0016125	sterol metabolic process	51	9.6E-23	3.8E-19
GO:0008203	cholesterol metabolic process	47	3.9E-21	1.5E-17
GO:0006732	coenzyme metabolic process	69	3.2E-20	1.3E-16
GO:0006631	fatty acid metabolic process	69	2.8E-13	1.1E-09
GO:0006790	sulfur metabolic process	45	7.8E-13	3.1E-09
GO:0006575	cellular amino acid derivative metabolic process	58	5.4E-12	2.1E-08
GO:0006641	triglyceride metabolic process	24	8.9E-11	3.5E-07
GO:0006639	acylglycerol metabolic process	24	9.5E-09	3.8E-05
GO:0006662	glycerol ether metabolic process	24	3.4E-08	1.3E-04
GO:0018904	organic ether metabolic process	25	3.4E-08	1.3E-04
GO:0019216	regulation of lipid metabolic process	28	7.7E-08	3.0E-04
GO:0043603	cellular amide metabolic process	20	3.3E-06	1.3E-02
GO:0046486	glycerolipid metabolic process	42	3.5E-06	1.4E-02
GO:0006006	glucose metabolic process	45	4.3E-06	1.7E-02
GO:0008206	bile acid metabolic process	11	1.1E-05	4.3E-02

a Bonferroni's correction was applied to adjust the P-values.

When the SNP genotypes were used together with the expression levels of the selected genes, for both of the traits, the accuracy of prediction was similar to that which was obtained using only the information of the selected genes. Thus, the observed accuracy did not substantially increase compared to that which was obtained using only the selected genes. It is likely that the influences of the selected genes were weakened when the gene expression data were used together with the SNP data, suggesting collinearity between, or ill-conditioning for, the SNP genotypes and gene expression levels when used as explanatory variables.

Overall, relatively high accuracy was observed when the expression levels of the TS (or SD) higher selected genes were used as explanatory variables, although statistical significance was only observed in a few cases. It is, therefore, possible that the phenotypic values of the present physiological traits are better explained by the genes that show differences in expression between tissues or individuals rather than by the genes that have greater genetic effects. Further investigations are necessary, including studies from the viewpoint of overcoming the redundancy of the SNP genotype and gene expression information used together as explanatory variables. The eQTL analyses indicated that specific SNPs can influence gene expression profiling and that this effect may contribute to the observed redundancy [8]. A SNP that is relevant to a trait is often an eQTL [38], and such SNPs have tissue-specific effects on gene expression [37]. Therefore, it may be necessary to remove the SNPs that strongly influence the gene expression profiles to overcome the redundancy issue.

### Final remarks

Predicting the phenotypes of quantitative traits as accurately as possible from information such as genome-wide genetic variations and gene expression in the absence of knowledge of causal variants is important in certain fields, including for the diagnosis of complex diseases and risk prediction for drugs in humans and for selection programs for crop and livestock species. To date, however, even in detailed diagnosis or prognostic risk prediction in humans, the sample size has ranged from fewer than 100 to a few hundred individuals [1], [2], [4], although van Wieringen et al. [3] integrated the data of approximately 100 to 250 individuals from three previous studies. Among mammals other than humans, there have been only two studies in mice [8], [18], which used gene expression data from liver samples from 132 to 440 animals.

In this study, we used available samples consisting of approximately 200 to 250 animals, which was also a limited sample size. However, we believe that accumulating such results is important because few relevant data are available worldwide. Because the gene expression data reflect effects that are not explained by the SNPs, integrating both genotypic and gene expression data as explanatory variables is a sophisticated approach whose validity should be investigated. In this study of physiological traits in mice, such as the glucose concentration and total cholesterol content, some profiles of the accuracy of phenotype prediction using SNP genotypic and gene expression information were determined, although few of these profiles showed statistical significance. Further investigations are required to confirm the current profiles of prediction accuracy and to overcome the redundancy of SNP genotypic and gene expression information as explanatory variables using additional data.
